# Cigarette package design: opportunities for disease prevention

**DOI:** 10.1186/1617-9625-1-2-97

**Published:** 2002-06-15

**Authors:** JR DiFranza, DM Clark, RW Pollay

**Affiliations:** 1 Department of Family Medicine and Community Health, University of Massachusetts Medical School, Worcester, Massachusetts, USA; 2 Faculty of Commerce and Business Administration, University of British Columbia, Vancouver, British Columbia, Canada

## Abstract

**Objective:**

To learn how cigarette packages are designed and to determine to what extent cigarette packages are designed to target children.

**Methods:**

A computer search was made of all Internet websites that post tobacco industry documents using the search terms: packaging, package design, package study, box design, logo, trademark and design study. All documents were retrieved electronically and analyzed by the first author for recurrent themes.

**Data Synthesis:**

Cigarette manufacturers devote a great deal of attention and expense to package design because it is central to their efforts to create brand images. Colors, graphic elements, proportioning, texture, materials and typography are tested and used in various combinations to create the desired product and user images. Designs help to create the perceived product attributes and project a personality image of the user with the intent of fulfilling the psychological needs of the targeted type of smoker. The communication of these images and attributes is conducted through conscious and subliminal processes. Extensive testing is conducted using a variety of qualitative and quantitative research techniques.

**Conclusion:**

The promotion of tobacco products through appealing imagery cannot be stopped without regulating the package design. The same marketing research techniques used by the tobacco companies can be used to design generic packaging and more effective warning labels targeted at specific consumers.

## Introduction

Tobacco manufacturers face a common challenge in marketing their cigarettes. Real differences between brands can be so small that blindfolded smokers may be unable to distinguish their usual brand from another [1]. To create a niche for their brand, manufacturers must strive to distinguish it from a field of hundreds. They do this by creating a brand image or brand personality [2]. The brand image is the sum of the product characteristics, advertising, promotion, package design and pricing. The design of the packaging plays a central role in the establishment of the brand image as the package is the touchstone to which all other advertising and promotional efforts relate.

While most consumers do not think of the package as advertising, this function of the package is well recognized by the marketing profession [3,4]. The package is the ultimate communication tool, the last step in the promotional process. The package should shape consumer expectations about the product in terms of quality and image. Packages are designed to be eye catching and attractive, to have visual impact both when seen alone, as in use, and when amassed in great quantities, as in large retail displays. When the package is displayed in the store, it is the sum of the product, the package, and the associated imagery that is purchased by the consumer. The idea that the consumer is actually purchasing something as intangible as an image may not have occurred to most consumers, but it represents a central premise of marketing.

Tobacco industry documents reveal that cigarette manufacturers are in the business of selling images and personalities [5-7]. They reveal a strategy of convincing "young starter smokers" that smoking a particular brand has "psychological benefits" in dealing with the emotional challenges of adolescence [8,9]. Brand images project the personality traits that adolescents want for themselves [8]. Youths who are insecure about their self image may take up smoking, in part, as a way of possessing or projecting the personality traits represented by the brand they choose [10]. Different brand images are used to appeal to mature smokers [11]. A successful advertising campaign will imbue the package with a personality that is meaningful to the targeted market segment. Consumers can then purchase the package and acquire the image for themselves to display to others. Cigarettes have been frequently described by marketers as "badge products." [12]. The package serves as a badge by bestowing upon the bearer the associated brand imagery. In the words of an industry insider,

*"cigarette packs... will have to work harder than ever, not only at the point of sale to provide increased 'stand out,' but also whilst in use to communicate the values of the brand to the consumer – to reassure and build loyalty. This is particularly so because of the role of cigarettes as 'badge products' with which the consumer identifies personally and which he uses to communicate his own identity to others" *[12].

One cigarette package designer states:

*"A cigarette package is unique because the consumer carries it around with him all day... it's part of a smoker's clothing, and when he saunters into a bar and plunks it down, he makes a statement about himself" *[13].

*"Smokers put their cigarettes in and out of their pockets 20 to 25 times a day. The package makes a statement. The consumer is expressing how he wants to be seen by others" *[14].

The package's utilitarian function of protecting the product is clearly secondary to the role of communicating an image. One marketing textbook comments:

*"package design is very important to advertisers because it is such an important part of brand image and product identity" *[3].

The brand's image is built by the package design and all of the accumulated associations created by advertising, promotions and sponsorships-associations of status, sophistication, glamour, fashion, and reward; associations of healthfulness and athleticism; associations of fun, excitement and risk taking; and associations of femininity, masculinity and sexual attractiveness [11]. Cigarette manufacturers devote a great deal of attention and expense to package design and this paper will share what we have been able to learn about this process by searching previously secret industry documents.

This study was undertaken to determine if there was evidence that cigarette package designs are targeted at children. We were also interested in the role of package design in the overall marketing strategy, the cost of implementing legislated changes in package design, any attempts to undermine the impact of the health warnings, the testing of "kiddie" packs containing 10 cigarettes, and the potential impact of generic packaging.

## Methods

Electronic depositories of tobacco company documents made available through product liability litigation were searched to identify documents relating to the design of cigarette packaging. The websites included those listed in Table 1. Search terms included packaging, package design, package study, box design, logo, trademark and design study. The first term searched at each site was package, followed by package design, tobacco package, cigarette box, packaging, package study, packaging study, and the additional terms listed above. The search was conducted between March 2000 and March 2001. Documents were retrieved electronically from the web sites.

**Table 1:** Websites searched for packaging documents

	This is the Phillip Morris Website. It loads searches fast and efficiently.
	This is the RJ Reynolds Website. This site has very lengthy search options, the newer site seems to have clearer search options than when our search was conducted. This link is no longer active. The following link was active as of 3-17-02
	The Council for Tobacco Research USA website. This site is similar to the RJ Reynolds site.
	This is the Center for Disease Control Website. It is relatively easy to navigate and user friendly. Many improvements have been made to make this site more accessible to the general public.
	The Tobacco Documents Online site has been improved since we conducted our search. It now offers selections from many tobacco sites.
	This website is still open although the link listed is no longer active. You can visit to search the same site for results. This site is maintained by Gene Borio a public health activist.
	The University of California site. This site yielded few useful results.
	The site of the US House of Representatives. The results are classified by Bates Number. Key word searches cannot be performed; each document would have to be read. Each document must be uploaded in tif (tagged image format). This is not only extremely time consuming but special software such as Paint Shop Pro or Adobe Photoshop is required to open the tif file.
	This is the House of Representatives' Website. This site is difficult to obtain usable results from.
	This site is maintained by the US House of Representatives, Committee on Commerce. It is not useful for searches, it requires the searcher to know the Bates Number. Documents must be downloaded in tif format.
	This site does not provide a searchable database. It provides links to the following sites.
	Philip Morris Inc. Tobacco Document Website
	
	Philip Morris Inc. Corporate Website
	
	R.J. Reynolds Tobacco Company Document Website
	
	R.J. Reynolds Tobacco Company Corporate Website
	
	Brown & Williamson/American Tobacco Company Document Website
	
	Brown & Williamson Corporate Website
	
	Lorillard Tobacco Company Document Website
	
	Lorillard Tobacco Company Corporate Website
	
	Tobacco Institute Document Website
	

We could not be certain that all relevant documents had been retrieved because it was quite common that the same search term yielded dramatically different results on the same website on different days. For example, a search term that yielded thousands of documents one day might turn up very little the next day. This suggests that either large numbers of documents were being removed from the websites, or that the key terms used to categorize documents were being changed. Because of the day to day variability in search results, repeated searches of individual websites were made using the same terms. The tobacco industry sponsored websites were often down for maintenance or updates or were unreachable.

The documents themselves are scanned images, rather than text files, and therefore cannot be searched for content. They are located using key words, which are assigned to the documents when they are posted on certain websites. Often searches retrieved documents with no apparent relation to the search term used. The skills and strategies of the people who assign the key words to the documents necessarily influences the quality of the search results.

All documents were printed out and reviewed by the lead author. A tally was kept of how many documents touched on various subjects. Quite often a subject was mentioned only tangentially; very few documents provided an in depth discourse of a particular topic. By analyzing the collected documents on each topic, a broad understanding could be obtained of how the tobacco industry works to design its packages.

## Search Results

Roughly half of the retrieved documents contained information that was relevant to this project and these have been posted on the web at *roswell.tobaccodocuments.org*. Among the 183 relevant documents, most dealt with the evaluation of proposed package designs, while others represented contracts and correspondence between the manufacturers and independent consultants. Table 2 presents a tally of the number of documents which touch on each of the listed topics. These will be discussed in detail below.

**Table 2:** The number of documents which mention or provide insight on each of the listed subjects

Subject matter	Number of documents
Design process	113
Consumer testing	103
Appearance of package	67
Product qualities/taste	64
Brand image	62
Perceptions of tar level	48
Test marketing	48
User imagery	48
Package graphics	47
Target market	43
Sensation transfer	23
Package configuration	19
Functionality	15
Findability/readability	13
Physical testing/manufacturing	12
Tachistoscope	10
Subconscious communication	3

**Figure 1:**
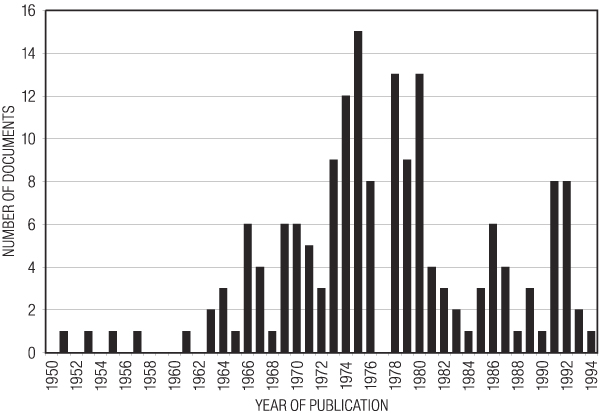
**Year of publication of the documents retrieved for this study**.

The period covered by the documents extended from 1951 to 1994 (Fig. 1). Seventeen documents were undated. The distribution of dates had a bell shaped distribution with a peak in the mid-seventies. We had expected that recent documents would be more common than those from 25 years ago. There was a curious absence of documents dated 1977. Enough information could be obtained from the documents to gain an understanding of how package design is conducted in this industry, but it is clear that the documents that were retrieved represent only a very small sampling of the documents that must have been generated in the course of conducting business. For example, there were no documents at all for many brands, although all have had packages designed. Many of the documents described a particular phase in what was outlined as a multi-phase project extending over many months or years, but no other documents could be located describing preceding or subsequent phases of these projects. Only a few documents contained guidance from the tobacco company to the agency contracted to design the package, although such documents must surely have existed for every contracted project. There were no documents reflecting management decisions regarding the final selection of competing package designs. No documents were found relating to the cost of implementing changes in package graphics. No documents discussed the targeting of minors, generic packaging, designing around the warning labels, or marketing experience with "kiddie packs."

### The design process

The ultimate design of the cigarette pack is the result of an involved and often lengthy collaboration between the tobacco company and contracted consumer research consultants [15-17]. The development of the famous Marlboro pack in the 1950's involved:

*"... 7 years of concurrent research on filter and box involving 27 Philip Morris research executives and supervisors alone; 25 scientists and technicians, many on the Company's staff, 8 outside consultants, 6 independent laboratories, 1 research organization and the busy fringe group of workers who receive no public credit beyond "and others" *[18].

Insight into the process of package design was provided by 113 documents. To understand how a cigarette package is designed, it is helpful to first consider its physical and marketing functions. The physical functions include protecting the cigarettes, maintaining freshness, keeping the cigarettes together and keeping loose tobacco out of the pocket or purse, while allowing easy access and generating minimum garbage with which the smoker must deal [19]. Fifteen documents mentioned the functional aspects of packaging. To fulfill its marketing functions, the package should work in conjunction with the media campaign to project a favorable image of the smoker, communicate the desired product attributes and imagery, generate quick brand recognition, stand out in a cluttered retail environment, appeal to smokers and motivate a purchase.

The package design process begins with a decision on the part of the company to proceed with either a design for a new brand or brand extension, or to redesign the pack of an existing brand. Reasons to redesign an existing pack might include a desire to modernize the design, to reinforce a new advertising strategy, to rekindle interest or excitement in an old brand, to signal changes to the product, to redirect the brand against a different target, or to stem the loss of market share resulting from changes in the package design of competitive brands [20-22].

*"Camel Filter is perceived to be a product with more strength than most other cigarettes. It is felt that this product image is inhibiting the potential of the brand... new packaging was developed with the intent of communicating a less strong product" *[21]*(RJ Reynolds (RJR))*.

*"Purpose: To determine if an alteration in package design would create a stronger flavor image for Doral." *[23]*(RJR)*.

### Product and user images

Whatever the motivation, the developmental process starts with a directive regarding the positioning of the brand in terms of the desired product imagery, user imagery, and the targeted smoker. Brand image was discussed in 62 documents, user imagery in 48, and the target market in 43. The user imagery is tied to the package's role as a badge. The user imagery should project a specific personality that can be attributed to the bearer of the pack. This brand personality offers the characteristics that the targeted smoker wishes to possess. Like choosing a wardrobe, by choosing a brand of cigarettes, the smoker purchases its personality and image for themselves. Since different personality images will appeal to different types of smokers, the tobacco companies tailor the brand personalities to the particular niche of smoker they are after, such as the blue-collar-male-full-flavor smoker, or the upscale-female-concerned-100 millimeter-mentholated-smoker.

*"Identify the type of personality target smokers would most admire in order to provide direction to the Agency for campaign development" *[5]*(Doral-RJR)*.

*"Psychological Benefit, A brand which is smoked by the type of person the prime prospect wants to be: A "man's man" who is macho and rugged, making him admired by men and irresistible to women. He wants to be in his late 20's, with extensive sexual exploits to his credit, athletic prowess, and savvy for finding the "right" places to go after dark. He is free, independent, and his job merely supports his outside activities. Life begins at five o'clock for him... For maximum image appeal, BY's package graphics should reflect the lifestyle and image which the prime prospect aspires to have... " *[24]*(BY is a code name for an unidentified RJR brand)*.

*"It [the red Marlboro pack] should be popular in its approach, have strong masculine appeal,... and be modern in its feeling, with no phony old world symbols of class or quality. Its modernism should be broadly popular-nothing avant-garde, yet nothing condescending" *[25]*(Philip Morris (PM))*.

*"Salem users will be perceived as natural and unpretentious, yet interesting individuals who are humorous/witty and are social catalysts within their peer group." *[7]*(RJR)*

*"Reactions to Stretch Pack among both franchise and competitive smokers indicated that desired user imagery was associated with the new pack (i.e., the Camel smoker as independent, self-assured, self-made" *[6]*(RJR)*.

*"The new Camel Lights Family packaging alternatives conveyed the desired user imagery (masculine, independent, assertive, self-confident, successful, strong) as well as demonstrated appropriate fit with Camel Family advertising and the "Beck" type smoker" *[6]*(RJR)*.

### Package Graphics

Working off the desired imagery, the consulting marketing research firm applies its experience to generate a variety of candidate designs. Package graphics were mentioned in 47 documents and the appearance of the package in 67. All aspects of the package design are important. Thus colors, graphic elements, proportioning, texture, materials and typography are tested and used in various combinations to create the desired brand image or personality [26].

*"Our new Philip Morris package is a result of over 2000 designs, all of which were carefully screened and tested... " *[27].

The proper selection of color is crucial to appealing to the correct target market. The selection of the color for the red Marlboro package was based on research conducted by the Color Research Institute of Chicago [18].

*"Red packs connote strong flavor, green packs connote coolness or menthol and white packs suggest that a cigarette is low tar. White means sanitary and safe. And if you put a low-tar cigarette in a red package, people say it tastes stronger than the same cigarette packaged in white" *[13].

*"In the case of menthol brands, green, apparently by definition, implies amount of coolness or menthol... The wood grain on Benson & Hedges symbolized richness, distinction, high class, wealth;" *[28]*(RJR)*.

Gold connotes quality, but at a premium price [29]. Of course, some colors appeal more to one gender than the other. Richer colors suggest a higher tar/full flavor cigarette, while pastels imply low tar/low flavor. The challenge is to communicate a low tar message without communicating low flavor.

*"However, other Ultra Lights (and MUL) smokers noted that the overall package appears to be "too light," limiting "taste" or "flavor cues" *[29]*(MUL stands for Merit Ultra Lights, PM)*.

*"Merit Filter (l): The "cream" background color was attention grabbing, especially in contrast with an array of white packages-However, the color was seen as having "yellow" or "beige" tones, suggesting a "strong" or "harsh" cigarette, to some and a "stale" cigarette, to others" *[29]*(PM)*.

*"Overall, Blue Packages were rejected by Merit and Merit Ultra Lights smokers as "too bright," "too bold," "too much of a statement" *[29]*(PM)*.

The font style is important to the impact of the design.

*"Package design should evoke graphic and visual cues representative of target lifestyle; i.e., strong vivid colors, (as on HBO/MTV, "Houston Knights") distinctive, unique typeface (as on neon bar signs, rock album covers, Mack truck grills)" *[30]*(Brand name 'Legend'-RJR)*.

Barely noticeable changes in the placement, orientation, number or thickness of stripes can all produce measurable changes in the impact of the design.

*"Vertical lines seemed to connote sleekness, length, compactness, versus masculinity, fatness, and thickness which is connected with the horizontal style" *[28]*(RJR)*.

*"Silver Background Pinstripes... Those favorable noted that the pinstriped background is more appealing than a plain white background, suggesting a higher quality image" *[29]*(Merit-PM)*.

Changes in the texture of the packaging wrapper, (paper, cardboard, foil, or plastic) are important [26,31]. Embossing creates the impression of a higher quality product.

*"Some consumers immediately noticed the embossed crest and were impressed by this detail. Once recognized as embossed, this feature was seen as reinforcing quality cues for the cigarette via attention to detail in its packaging" *[29]*(Merit-PM)*.

### Package configuration

Manufacturers have experimented with a wide variety of novel shapes and sizes for cigarette packs, including cylinders, triangles, semi-circles and ovals [32,33]. Package configuration was mentioned in 19 documents and the functionality of the pack in 15. One document includes drawings for more than 60 ideas for completely novel packaging configurations [33]. Different mechanisms for opening the pack have been explored, such as "A PEZ-LIKE FLIP OPEN TOP," hinged lids, slide out drawers, and re-closable flaps with Velcro or snaps [26]. A variety of mechanisms for dispensing cigarettes one at a time have been investigated, including individually wrapped cigarettes on a roll that are torn off as needed. Packs that break apart into two or more smaller packs have been evaluated. Packs fashioned from plastic and aluminum have been considered to better preserve freshness, but these nonbiodegradable materials were rejected due to environmental concerns [34,35]. From the uniformity of current cigarette pack configurations it is evident that none of these novel approaches have had lasting appeal in the USA.

### Physical Testing

The physical functions of the pack are evaluated in the company research facilities. Tests are conducted to measure the crush resistance and moisture retention properties of alternative packaging materials [34]. Twelve documents concerned physical testing or manufacturing issues.

### Consumer Testing

The evaluation of the marketing performance is more involved and is commonly contracted to outside firms. Candidate designs are subjected to a variety of qualitative and quantitative tests to determine which best meets the design criteria [36,37]. Consumer testing was mentioned in 103 documents. Philip Morris, when seeking more women smokers for its Parliament brand, changed the package design to reduce its masculinity and raise its femininity and tested the prototypes on 600 subjects [38].

The first evaluations are made with color drawings of the proposed designs [15]. Due to cost considerations, actual life size three dimensional, mock-ups of the packs are not generated until the field of contestants has been narrowed down to only a few [21,39]. Full scale mock-ups are required to evaluate the texture and feel of the pack, as well as its physical functions.

The initial evaluations of the designs are conducted with either individual consumers or small homogeneous focus groups. The subjects in these studies are carefully chosen based upon demographic considerations and their current smoking preferences [40].

*"Interviews were conducted among men who smoke 85 mm non-menthol filter flavor cigarettes" *[41]*(RJR)*.

Subjects are asked to identify positives and negatives for each design to gain insight into which of many possible designs might be most appealing to targeted consumers [42,43].

*"Projective questions were asked about the packs in order to ascertain the particular images and appeals evoked by each" *[44]*(Camel-RJR)*.

The testing will often begin with open questions to evaluate consumers' spontaneous impressions of the packages and their contents. For example, subjects might be asked "what type of person would smoke this brand?" Open ended questioning is followed by more structured testing in which the ability of each design to meet the design objectives is evaluated. For example, if the goal of the design change is to communicate a lower tar message, alternative designs will be compared on this performance criteria. In practice, each design is simultaneously rated on its ability to communicate dozens of attributes concerning both the product image and the user image. Table 3 lists a variety of characteristics used to describe the visual impact of the pack. Table 4 lists characteristics used to evaluate the user image generated by each design, and Table 5 lists qualities relevant to the product image, that is, the projected qualities of the cigarette.

**Table 3:** Qualities used by Philip Morris and RJ Reynolds to rate cigarette packages according to appearance and functionality [19,45-47]

	Appearance
Rich looking/smart/elegant	Liked the colors
Different	Simple/neat
Looks cold/refreshing/clean	Eye-catching/bold
Bright	Attractive
Happy	Modern and up-to-date
Ordinary	Plain
Similar to others	Cheap looking
Dull/drab	Unattractive
Messy/chopped up/gaudy/cluttered	

	Functionality

Was easier to open	Did a better job keeping tobacco out of your pocket or purse
Was easier to close	Did a better job of keeping cigarettes from spilling out
Protected the cigarettes better	Did a better job of keeping cigarettes fresh
Was more pleasing to look at	Did a better job of keeping cigarettes from being crushed
Felt more comfortable in your hand	Was more different from other cigarette packages
Fit better in your pocket or purse	

**Table 4:** Selected attributes used to rate user imagery generated by Philip Morris Company package designs [48,49]

Lively	Youthful	Exciting
More Sociable	More Spontaneous	More Active
More Natural/Down to earth	More Warm/Caring	More Self Confident
Enjoys the outdoors more	More Feminine	Pushy
More Modern	More Stylish	More Spirited/Fun
Higher income	More Sophisticated	Sensitive/caring
Friendly	Does things on the spur of the moment	

**Table 5:** Attributes used by Philip Morris to rate package designs according to implied product imagery [47,49]

Good taste	Low tar	Low nicotine
More effective filter	More modern	Higher quality
Costly	Smart	More improved
Richer tobacco	Better flavor	Freshness
Good value	A high quality cigarette	Smooth tasting cigarette
More satisfying	Lower tar	Lighter tasting
Stronger	Harsher	Milder
Cooler	More refreshing	More menthol taste

### Consumer Research Methods

Tests may be conducted by stopping people in shopping malls, by telephone, or subjects may be invited to a central facility. Potential designs are tested in either a monadic or paired comparison design [23,50]. In monadic tests, subjects are shown one design at a time and asked to rate the pack on characteristics such as those listed in the tables. Subjects are given either opposite word pairs and asked to choose between the two, or are asked to rate each quality on a "thermometer" scale from 0 to 100 [45,51]. In a paired comparison, subjects are shown designs side by side and asked to rate one against the other. This is done to compare a few final contestants, to compare a new design to the current design, or to compare a new design to a competitor's pack. Subjects' reactions to the pack designs are broken down statistically according to gender, income, region, and current brand preference [48]. Sophisticated statistical analyses may be performed. Through this process, it is determined which of the candidate designs best communicates the desired product and user imagery.

*"The Timer Yellow Flying M design was preferred to the Modified Wedge execution both overall... and in terms of having "better tasting cigarettes"... and being most suggestive of a "scientific breakthrough"... The two package designs tied... with regard to perception of "tar level" (Statistical data omitted) *[52]*(Timer-PM)*.

*"In a few instances, the new full flavor pack was seen as containing cigarettes which are lighter and smoother. In terms of pack image, the new full flavor, lights and 120's packs produced a more stylized smoker profile (classy, elegant, distinctive, stylish, trendy) than the current packs" *[53]*(Virginia Slims-PM)*.

*"In addition, the current packaging communicated the desired product imagery (i.e. smooth, satisfying, cool, refreshing) and user imagery..." *[30]*(Code name LF-RJR)*.

*"Whereas the "Diagonal Stripe" smoker would most likely be a young debonair man, the typical "Split Red/Gold" smoker would most likely be an average housewife type, although there appears to be some "businessmen or business-women" connotation to this package. The typical "Red Pack" smoker would appear to be a rugged type man" *[54]*(Mark VII-PM)*.

*"The current packaging communicated a more active, spirited, sociable, warm/caring, self-confident, modern and fun-loving user image" *[47]*(Philip Morris brand-PM)*.

*"The blue package would be consistent with, and would enhance an image of a product with full flavor, perhaps too strong – even harsh and irritating, appealing to young, fashionable men who consider themselves continental and vivacious. The white pack, on the other hand, would contribute an image of mildness, smoothness, appealing to older, conservative, down to earth women. The choice, then, becomes one of picking the package that is more consistent with the over-all marketing positioning the product is to be given" *[55]*(Parliament-PM)*.

The amount of product and user imagery that can be communicated without text or even pictures is impressive. Design details deliver these 'goods'.

### Subconscious Advertising

Three documents discussed how the package design communicates to consumers through subconscious processes. The utility of objective testing is felt to be limited because the package design works through subliminal processes.

*"We would probe the conscious attitudes on the new designs even though we may feel a package operates subconsciously and connotatively" *[28].

*"We are cognizant of the fact that at least part of the impact of a package design operates on a subconscious level" *[28].

*"On the first level a package serves to reinforce the brand's advertising in establishing a certain brand image or set of connotations, and in so doing it operates on a subconscious level. That is, the fact that it does this is not readily apparent to the consumer" *[28].

Actual text on cigarette packages is quite limited. Most of the imagery and communication of ideas to the consumer is achieved through the use of colors, shapes and textures. Often the consumer is not aware that this process is occurring. For example, the color white is commonly used on low tar brands which were created to allay smokers of their health concerns. White is a color commonly associated with health care facilities. Nurses uniforms were traditionally white. Through the use of the color white on packages of low tar cigarettes, manufacturers can capture the consumer's stored associations of white with health.

### Tachistoscope

Mock-ups of the narrowed field of designs are manufactured to allow for further testing. The ability of each design to quickly communicate the brand name is tested with a tachistoscope [46,49,56-58]. Ten documents mentioned the tachistoscope, a device which allows researchers to expose subjects to package designs under controlled conditions of lighting, size, distance and exposure times [58]. Exposures start at a few milliseconds. The subject is asked to describe any features of the pack that can be identified. The Marlboro pack was identified with only a four millisecond exposure [58]. Exposures are gradually lengthened until all features of the pack can be identified. The percentage of subjects who can read the brand name with exposures of 1/10, 1/8, 1/4 and 1/2 second are measured. The readability of the brand name can be affected by font size, font style, color, color contrast, text orientation, and design elements which compete for visual attention.

*"Four of the five Cambridge packages which were audited (in both the Current and Proposed formats) were able to communicate the Cambridge name to over 80% of the smokers within 1/8 of a second" *[46]*(PM)*.

### Findability

The "findability" of the pack may be quantified using a simulated shelf display in which the pack is positioned with several other brands and subjects are asked, after a 3 second exposure, to recall where it was located [46,49]. In-store tests of pack displays have been conducted with hidden cameras and one-way mirrors used to track shoppers' eye motions [18,59]. Carefully controlled experiments have been conducted in chains of convenience stores to determine the independent impact of displays and discount offers [60]. Manufacturers strive for "stand out," the package should stand out from the clutter of competing brands and be easily seen in a display rack. Thirteen documents concerned the findability or readability of package designs.

### Sensation Transfer

Perhaps the most intriguing tests are the widely used "sensation transfer" tests [19,61-63]. These were mentioned in 23 documents dating back to 1967. Subjects are given two packs with different designs containing identical cigarettes. They are asked to smoke one cigarette from each pack and to then rate the two cigarettes on a variety of criteria, such as taste, smoothness, after-taste, and quality. Surprisingly, the subjective evaluation of the cigarettes can be significantly impacted by the package design. In one test, 36% of women judged the cigarette removed from the current package to have good taste compared to 19% for an identical cigarette removed from the test package [61]. In another study, men preferred the cigarette in the "ice pack" (61%) over an identical cigarette in the "green pack" (22%) [62]. The influence of the package design on the subjective qualities of the cigarette are such that when an objective rating of the cigarette qualities is desired, the test cigarettes are all placed in standardized white packaging [64].

### Cannibalism

Manufacturers are always interested in increasing their market share by maintaining their "franchise", smokers who already smoke their brand, while attracting "trials" by smokers of the brands of their competitors [7,65]. Designing a pack to have maximum appeal to smokers who do not currently smoke the brand risks alienating current franchise smokers and particular care is given to avoid this scenario [7,66]. There is also concern that a change might result only in the cannibalization of market share of the company's other brands resulting in no overall benefit to the company [6]. The reactions of current franchise smokers are evaluated by asking them to compare and rate the proposed pack with their current pack and by asking if they would consider switching to another brand if the proposed changes were to be implemented.

*"Smokers of WINSTON King and SALEM King are extremely familiar with the package design of their usual brand. Even when the current package was tested against changes only in the color of the foil (from silver to gold) and the closure seal (from blue to red or green), about 100% recognized the currently marketed package. These results and past experience with package changes (most have resulted in sales losses-exceptions are Marlboro 100's and L&M) say that we should proceed with caution in introducing these package changes into the marketplace" *[67]*(RJR)*.

The "trial" potential of new designs are tested by studying the reactions of smokers who fit the target but who currently smoke the competition's brands in that market niche [46,68,69].

### Test Marketing

If the proposed pack performs as well as the current pack with franchise smokers, a regional test-marketing might be performed next [70,71]. Test marketing was mentioned in 48 documents. It is done primarily to detect any potential negative effects on brand sales. Test marketing requires a significant investment as actual packages containing cigarettes must be manufactured. Shipments of the current package are stopped in carefully selected test markets, typically a major city, and replaced with shipments of the new design [42,70]. The introduction of the new package design typically coincides with a new advertising campaign in the test area, complete with in-store displays. Displays are designed and tested to attractively present the packs. Contractual arrangements are set up with retailers whereby the company's brands are given prominent display for a set amount of time [72]. Test market shipments are monitored and compared to simultaneous changes in the brand's market share in other regions. Changes in state or local tobacco excise taxes can disrupt market tests [73]. If the test market results show no decline in sales, it is likely that a national roll-out will follow.

### Health Concerns

There was ample evidence that the package design is used to convey an impression of lower tar and nicotine delivery. Only two documents actually used the word "health" in their evaluation of package designs. However, perceptions of tar level were mentioned in 48. Reductions in tar levels are typically associated with reductions in the strength of the taste of the smoke. A very common objective of package designers is to simultaneously communicate messages of low tar and full flavor. This is quite a challenge as design elements that communicate low tar bring out connotations of low flavor, while elements that communicate full flavor often imply high tar. To the degree that the low tar/low nicotine message is meant to assuage smoker's health concerns it is clear that the package design plays an integral role in this strategy [11].

*"The "Green Line" design was the most effective in connoting lowered tar and nicotine,.." *[74]*(Salem-RJR)*.

*"Communicate product perceptions of "low tar" as well as Virginia Slims' packaging" *[75]*(Salem-RJR)*.

User imagery is tested to determine if the package design would be used by a smoker who is "concerned about health" [76]. While the following quote does not specifically address health, it raises concern that subconscious processes may be at work to undermine the conscious health arguments against smoking.

*"And the fact that sensation transfer from package to contents is usually made on a subconscious level, weakens any information which is received via conscious methods. It is my belief that stimulating the subconscious desires is much more effective than concentrating on the conscious ones. If a person is aware of a stimulus, logical thinking may rationalize away the need. However, on a subconscious level this same human being may be strongly motivated to satisfy the need aroused" *[77]*(RJR)*.

## Comment

We found no evidence in the documents that we were able to locate, to indicate that cigarette manufacturers target children with their package designs. This does not represent proof that such targeting does not occur. A different search strategy may have unearthed more documents. It is possible that potentially embarrassing documents may have been destroyed, or withheld. The fact that we were able to locate more documents from the 1970's than from subsequent decades suggests that there has been a selective release of documents. The documents analyzed here certainly do not represent all of the manufacturers' activities concerning package design.

Cigarette manufacturers carefully research their package designs to ensure that they project the desired product and user images and personalities. Wide ranging restrictions on the advertisement and promotion of tobacco products have been implemented, but in every case the package design has been exempted [78-80]. If the goal of these restrictions is to curtail the promotion of tobacco products, tobacco companies must be prevented from using the package to conjure up enticing and reassuring imagery. To this end, regulations which would require "generic" or "plain" packaging, devoid of promotional qualities, have been proposed in Canada and Australia (Table 6) [81].

**Table 6:** A Definition Of Generic Packaging Offered By The Canadian Cancer Society [81]

The package shall have an unattractive base color inside and out and shall have no writing or markings other than the brand name, warnings required by law, tax stamp information and bar codes.
The location of the name shall be restricted, possibly to one small end of the package.
The brand name shall be printed in black ink in a standardized font and type size.
Trademarks shall be prohibited.
All packages would be standardized in terms of size, packaging materials, method of opening, embossing and texture.
A minimum package size of 20 cigarettes would apply.
The same requirements would apply to cartons.
Any decorative attachments would be prohibited.
Plain packaging would apply to all forms of tobacco.
All brands would look the same, distinguished only by the name of the brand printed in a standard plain font.

Experimental evidence demonstrates that the cigarette package is capable of generating positive user imagery even without additional promotional communications. In New Zealand, 568 youths in 80 focus groups were shown packs of cigarettes [82]. Solely on the basis of the package design, without being exposed to advertising, the youths were able to construct a brand personality for the American packs [82]. By contrast, no brand imagery was associated with generic packs. As long as manufacturers have the package design to work with, they will be able to create brand images which appeal to consumers.

The potential impact on adolescents of requirements for generic packaging has been evaluated [82-84]. Australian youths were asked to comment on a generic pack of cigarettes with much larger and effective health warnings. Their comments included, "the image is lost", "they take away the company look", "wouldn't be cool with these boxes", "I would be embarrassed to hold them", "people would feel like rejects if they carried these", "they look yucky", "are these real?", "its saying don't buy me" and "I wouldn't buy them" [83]. In another study, youths were shown generic packs that were beige with black lettering and displayed only the name of the brand, the UPC bar code, health warnings, and tar and nicotine levels [JR d'Avernas et al. unpublished manuscript]. Youths found the health warning on plain packs to look more serious and to be more noticeable, even though the warnings were the same on both types of packs. The generic packages were rated as less... attractive, exciting, modern, for young people, expensive looking, colorful, cool and less likely to turn young people on to smoking. Youths in New Zealand perceived generically packaged brands to be "dull and boring." [82]. In another study from New Zealand, youths demonstrated greater attention to, and recall of, health warnings when generic packaging was used [84]. Youths learned and recalled the brand information before other elements of the package such as the health warning. As the amount of brand information increased, the impact of the health warnings decreased. Thus the promotional aspects of package design blunted the impact of the health warnings.

The package can be thought of as a billboard upon which the tobacco industry and health community present competing messages. The promotion of the product is conducted through the skilled application of colors, textures, designs, logos, fonts and text to create positive imagery. This is followed by extensive consumer testing. The health message is typically presented in wordy text delivered in a plain black font on a white background with no testing at all. Under these circumstances, the health message can't compete. One clear lesson from this study is that if the public health message is to succeed, the same effort must be put into the design of warning labels and generic packaging as the tobacco companies put into designing the cigarette pack.

The process of designing generic packaging and more effective warning labels could begin by establishing consumer targets. Just as particular brands are targeted at particular demographic or marketing niches, warning labels could also be tailored and targeted by brand niche. For example, drawing on stages of change theory, the warning labels for the low tar and nicotine brands smoked by "hi-fi" (high-filtration) smokers who have health concerns could be tailored differently than those for "high tar/full-flavor" smokers [85]. Warnings for male predominant brands would include impotence, while those for female brands could stress pregnancy complications, osteoporosis and cervical cancer. Mentholated brands could emphasize the impact of smoking on minorities.

Following the example of the tobacco industry further, warning labels should be consumer tested using a variety of fonts, colors, shapes and borders. If package attributes can be selected to best communicate "masculine, independent, assertive, self-confident, successful, strong," or "active, spirited, sociable, warm/caring, self-confident, modern and fun-loving" it should be possible to select the fonts, colors and borders that best communicate "dying with lung cancer", "crushing chest pain", or "suffocating with emphysema" [6,47]. Different colors, shapes, borders or fonts might be most appropriate for each type of warning. For example, the impotence warning might be more effective with a nice pink background and lacy border. Visual elements of the warning labels could be carried over into mass media communications to expand and reinforce the message.

Warning labels and generic packs should be tested for impact, first with focus groups and then with quantitative testing. The goal in designing a generic pack should not be to design an ugly pack, but one which reinforces the message of the health warnings. Generic package designs could be rated as to their ability to counteract the socially desirable images created by past advertising. For example, generic packs could be selected based on smokers' assessments as to which would most likely be chosen "by a loser", "by someone who has bad taste in clothes", or "by a boring person with no friends".

Sensation transfer tests could be conducted with competing generic package designs to determine which package makes the cigarette taste the least rewarding.

The design of cigarette packages is central to the tobacco companies' efforts to promote tobacco use. If the goal is to discourage tobacco use, the package design should be eliminated as a source of positive imagery. The same marketing research techniques that have been used to promote tobacco use can be enlisted in the fight against this addiction and the diseases and suffering it causes.

## Competing interests

The authors declare that they have no competing interests.
